# Acquired Palmoplantar Keratoderma Revealing Primary Biliary Cholangitis: Therapeutic Response to Ursodeoxycholic Acid

**DOI:** 10.7759/cureus.117007

**Published:** 2026-09-27

**Authors:** Hajar Dahmani, Ouiame El Jouari, Sabrina Oujdi, Salim Gallouj

**Affiliations:** 1 Dermatology Department, Faculty of Medicine and Pharmacy, Abdelmalek Essaadi University, University Hospital Mohammed VI, Tangier, MAR

**Keywords:** acquired palmoplantar keratoderma, cholestasis, dermoscopy, primary biliary cholangitis, ursodeoxycholic acid

## Abstract

Acquired palmoplantar keratoderma (PPK) may be associated with drug exposure, inflammatory or infectious dermatoses, malignancies, and systemic disorders. Although several systemic associations have been described, its occurrence in primary biliary cholangitis (PBC) appears to be exceptionally rare. We report the case of a 36-year-old woman with no significant medical history who presented with a three-month history of diffuse, non-pruritic palmoplantar keratoderma refractory to topical therapies. Clinical examination revealed erythema with multiple punctate yellowish hyperkeratotic lesions, some coalescing into plaques, involving the palms and soles. Dermoscopy showed a pinkish background with multiple yellowish, occasionally confluent, plaque-like scales and no visible vascular pattern. Hematologic, renal, thyroid, and infectious investigations were unremarkable. Liver biochemical tests, however, revealed a cholestatic pattern, and anti-mitochondrial antibodies were positive. PBC was diagnosed based on the combination of cholestatic liver biochemistry and anti-mitochondrial antibody positivity. Treatment with oral ursodeoxycholic acid was followed by progressive improvement of the palmoplantar lesions, with marked clearing and normalization of liver biochemical tests after three months. This observation adds to the very limited literature linking acquired PPK with PBC and highlights the importance of investigating an underlying systemic disorder in otherwise unexplained or treatment-refractory acquired keratoderma.

## Introduction

Palmoplantar keratodermas (PPKs) comprise a heterogeneous group of disorders characterized by excessive keratinization and thickening of the skin of the palms and soles. They are broadly classified into hereditary and acquired forms. Hereditary PPKs generally have an earlier onset and may be associated with a positive family history, whereas acquired PPKs typically develop later in life, in the absence of a relevant family history, and are usually related to an identifiable underlying condition [1,2]. Acquired PPK has been defined as a non-hereditary, non-frictional hyperkeratosis of the palms and/or soles involving ≥50% of the surface of the affected acral areas, with or without clinical and histologic inflammation [1]. It encompasses a wide spectrum of etiologies.

Acquired PPK may be associated with chemical exposure, nutritional disorders, medications, inflammatory dermatoses, infections, malignancies, and systemic diseases [1]. Among systemic disorders, recognized associations include hypothyroidism, myxedema, chronic lymphedema, and circulatory disorders [1]. By contrast, associations with autoimmune hepatobiliary diseases are uncommon. Primary biliary cholangitis (PBC), in particular, has been associated with acquired PPK in only two previously reported cases [3,4].

Primary biliary cholangitis is a chronic autoimmune cholestatic liver disease characterized by progressive immune-mediated injury to the small intrahepatic bile ducts and a strong association with circulating antimitochondrial antibodies [5]. It predominantly affects women and may be accompanied by several extrahepatic autoimmune manifestations. Its dermatologic manifestations most commonly include pruritus, xerosis, hyperpigmentation, and xanthomatous lesions, whereas acquired PPK is distinctly unusual [5,6]. Therefore, PBC may be considered in otherwise unexplained acquired PPK after more common causes have been excluded. The pathophysiological mechanism linking PBC to palmoplantar hyperkeratosis remains unknown and has not been systematically explored. We report the case of a 36-year-old woman with acquired palmoplantar keratoderma leading to the diagnosis of previously undiagnosed PBC, with complete clearance of the cutaneous lesions following ursodeoxycholic acid therapy, and discuss the potential pathophysiological links between these two conditions in light of the available literature.

## Case presentation

A 36-year-old woman with no significant personal or family medical history presented to our dermatology department with a three-month history of non-pruritic palmoplantar thickening. She reported no relevant occupational or chemical exposure and was not taking any oral medications. She had previously received empirical treatment with topical keratolytics, topical antifungal agents, topical corticosteroids, and topical retinoids, without clinical improvement.

Clinical examination revealed bilateral and symmetrical, non-transgradient yellowish palmoplantar keratoderma with moderate underlying erythema. The palmar involvement was predominantly diffuse (Figure 1), whereas the plantar involvement consisted of punctate hyperkeratotic lesions, some coalescing into plaques over pressure-bearing areas (Figure 2).

**Figure 1:**
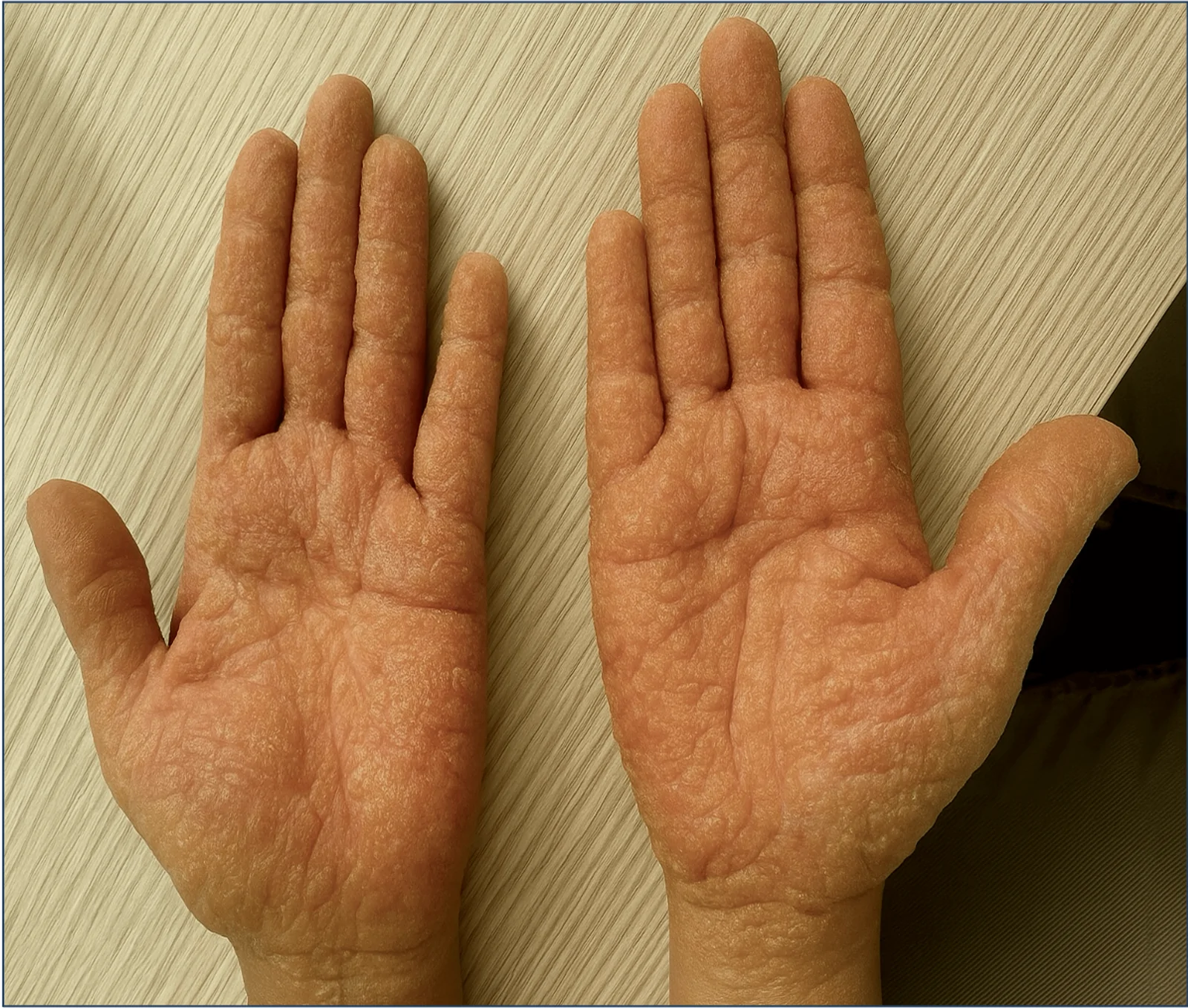
Diffuse yellowish palmar keratoderma with mild underlying erythema.

**Figure 2:**
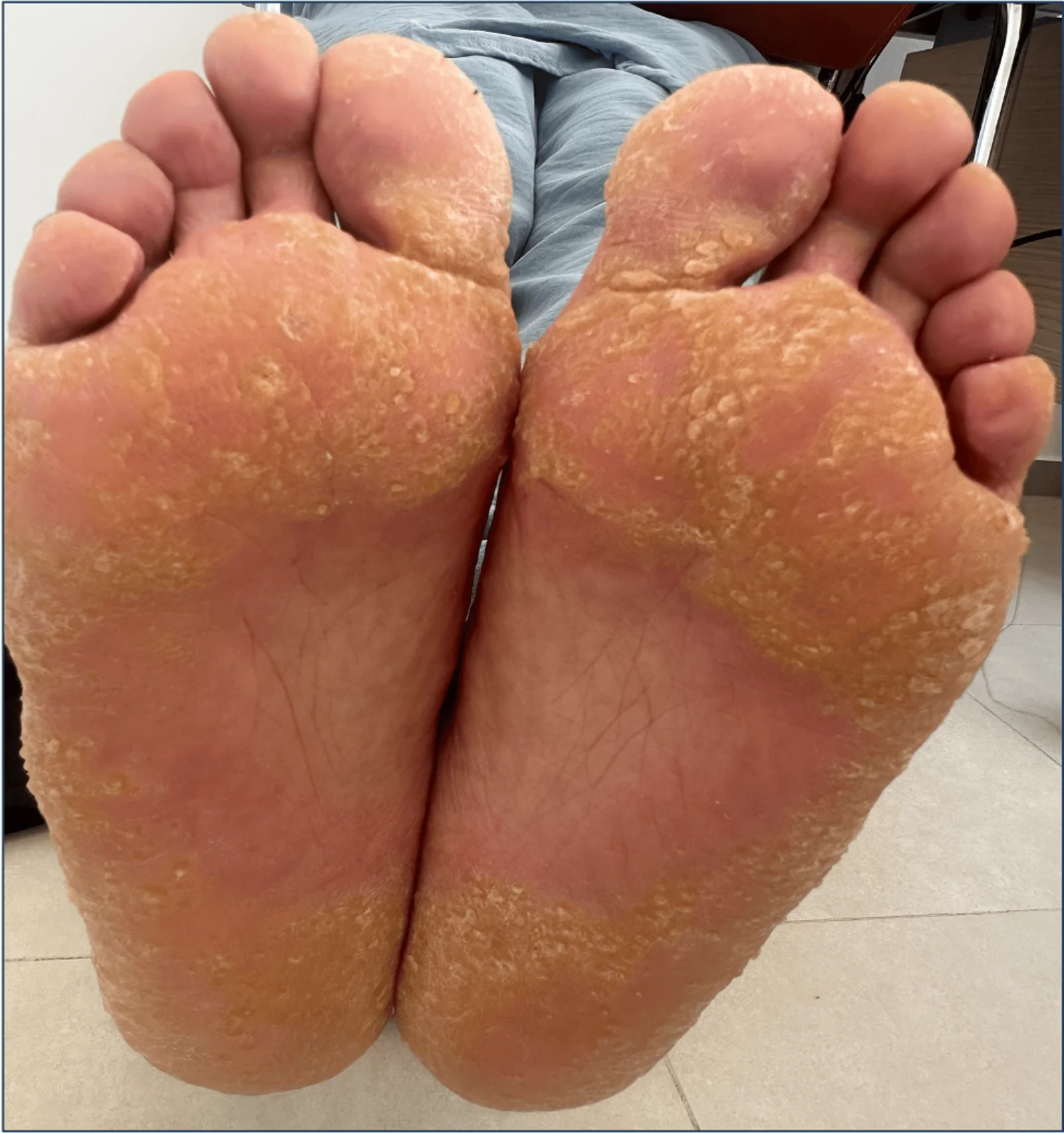
Plantar erythema with thick yellowish hyperkeratotic plaques, predominantly involving pressure-bearing areas.

Dermoscopy performed with a DermLite DL5 (DermLite LLC, Aliso Viejo, USA) in polarized mode showed a pinkish background with multiple round-to-oval yellowish areas covered by patchy plaque-like scales, occasionally confluent, with no visible vascular pattern and without disruption of the dermatoglyphic skin markings (Figure 3).

**Figure 3:**
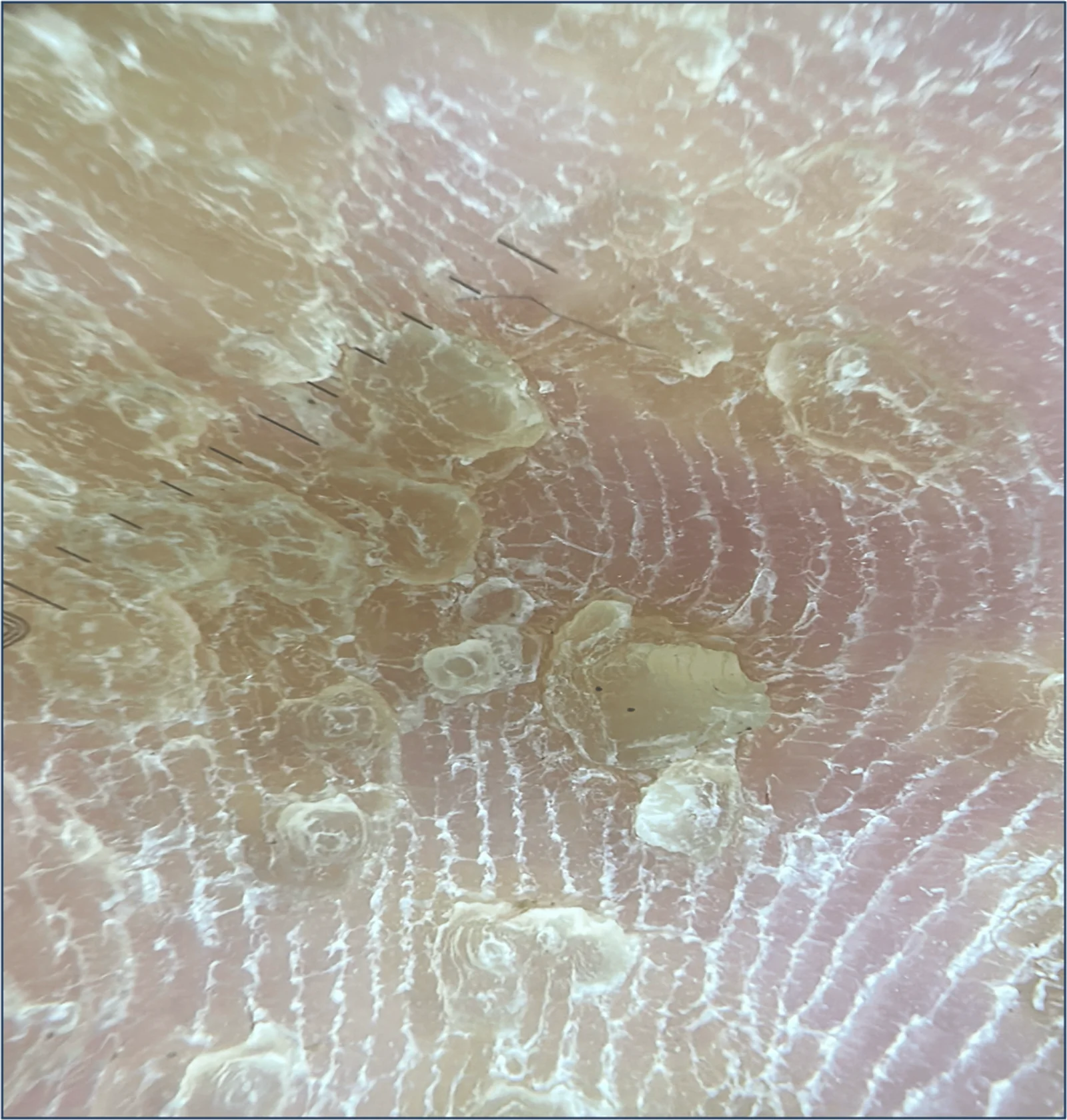
Polarized dermoscopic appearance (DermLite DL5): pink background with multiple yellowish circular areas covered by patchy, sometimes confluent scales, with no visible vascular network.

No other cutaneous lesions were observed, and there were no mucosal symptoms or systemic signs on clinical examination. She had no history of similar palmoplantar lesions during childhood or adolescence, and there was no family history of palmoplantar keratoderma. No parental consanguinity was reported. No associated nail, hair, or dental abnormalities suggestive of a hereditary or syndromic palmoplantar keratoderma were identified. She was premenopausal and reported no relationship between the lesions and water exposure. There were no clinical features suggestive of nutritional deficiency or underlying malignancy and no history of chronic inflammatory dermatosis.

Direct mycological examination and bacterial culture were negative. A skin biopsy was proposed but declined by the patient. Laboratory investigations revealed a normal complete blood count, renal function, thyroid-stimulating hormone level, and total bilirubin level. Serologic testing for human immunodeficiency virus (HIV) and syphilis was negative, and antinuclear antibodies were negative.

Liver biochemical tests, however, showed abnormalities consistent with a predominantly cholestatic pattern, with an alkaline phosphatase level of 200 U/L. The patient was referred to the hepatology department for further evaluation. Abdominal ultrasonography was unremarkable, with no biliary duct dilatation or evidence of extrahepatic obstruction. Further serologic testing revealed positive anti-mitochondrial antibodies (AMA) at a titer of 1:120 (Table 1).

**Table 1:** Laboratory investigation results. TSH: thyroid-stimulating hormone; AST: aspartate aminotransferase; ALT: alanine aminotransferase; GGT: gamma-glutamyl transferase; TPHA: treponema pallidum hemagglutination assay; VDRL: Venereal Disease Research Laboratory; Ag/Ab: antigen/antibody.

Laboratory test (unit)	Result	Reference range
Hemoglobin (g/dL)	13.4	12-16
Leukocyte count (/mm³)	7,500	4,000-10,000
Platelet count (/mm³)	298,000	150,000-400,000
TSH (mIU/L)	2.06	0.4-4.0
Urea (g/L)	0.26	0.15-0.45
Creatinine (mg/L)	8.7	6-11
AST (U/L)	87	6-25
ALT (U/L)	94	6-25
GGT (U/L)	93	5-35
Alkaline phosphatase (U/L)	200	35-95
Total bilirubin (mg/dL)	0.7	0.2-1.0
Antinuclear antibodies (ANA)	Negative, 1:20	<1:80 negative
TPHA/VDRL	Nonreactive	Nonreactive
HIV-1/2 Ag/Ab	Negative	Negative
Anti-mitochondrial antibodies (AMA)	Positive, 1:120	<1:40 negative

The diagnosis of primary biliary cholangitis was established based on the combination of cholestatic liver biochemistry and AMA positivity. Oral ursodeoxycholic acid was initiated at a total dose of 900 mg/day (approximately 14 mg/kg/day), administered as 300 mg three times daily for the treatment of PBC. Previous skin-directed treatments had been discontinued, and no concomitant dermatologic therapy was prescribed.

At follow-up, progressive improvement of the palmoplantar lesions was observed. After three months of ursodeoxycholic acid (UDCA) therapy, complete clearance of the lesions was achieved (Figures 4, 5), together with normalization of liver biochemical tests (aspartate aminotransferase (AST): 22 U/L, alanine aminotransferase (ALT): 24 U/L, gamma-glutamyl transferase (GGT): 31 U/L, and alkaline phosphatase: 78 U/L). The clinical response was sustained over 18 months of follow-up, with no relapse of the palmoplantar keratoderma.

**Figure 4:**
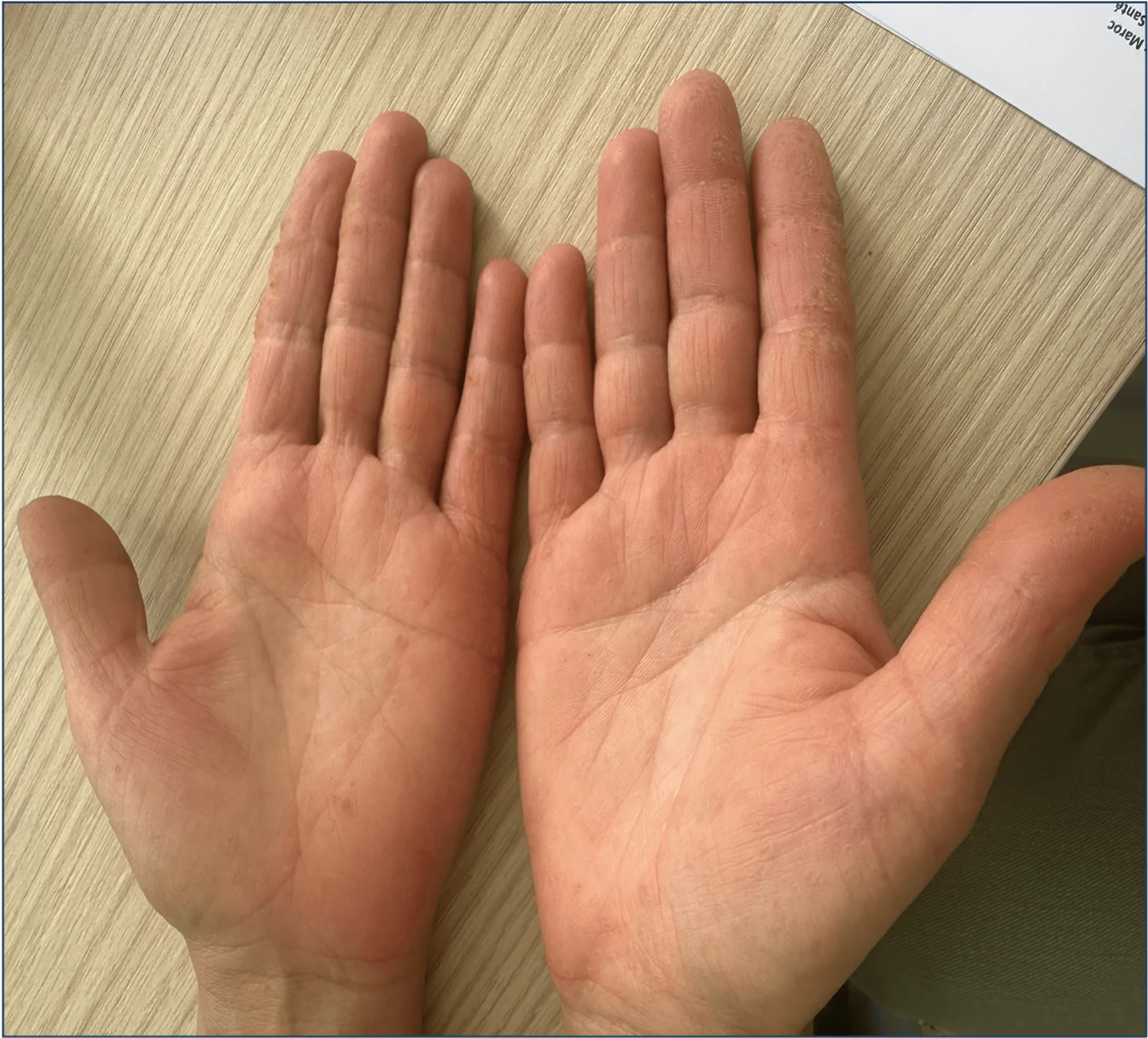
Clinical improvement of the palms three months after initiation of ursodeoxycholic acid therapy.

**Figure 5:**
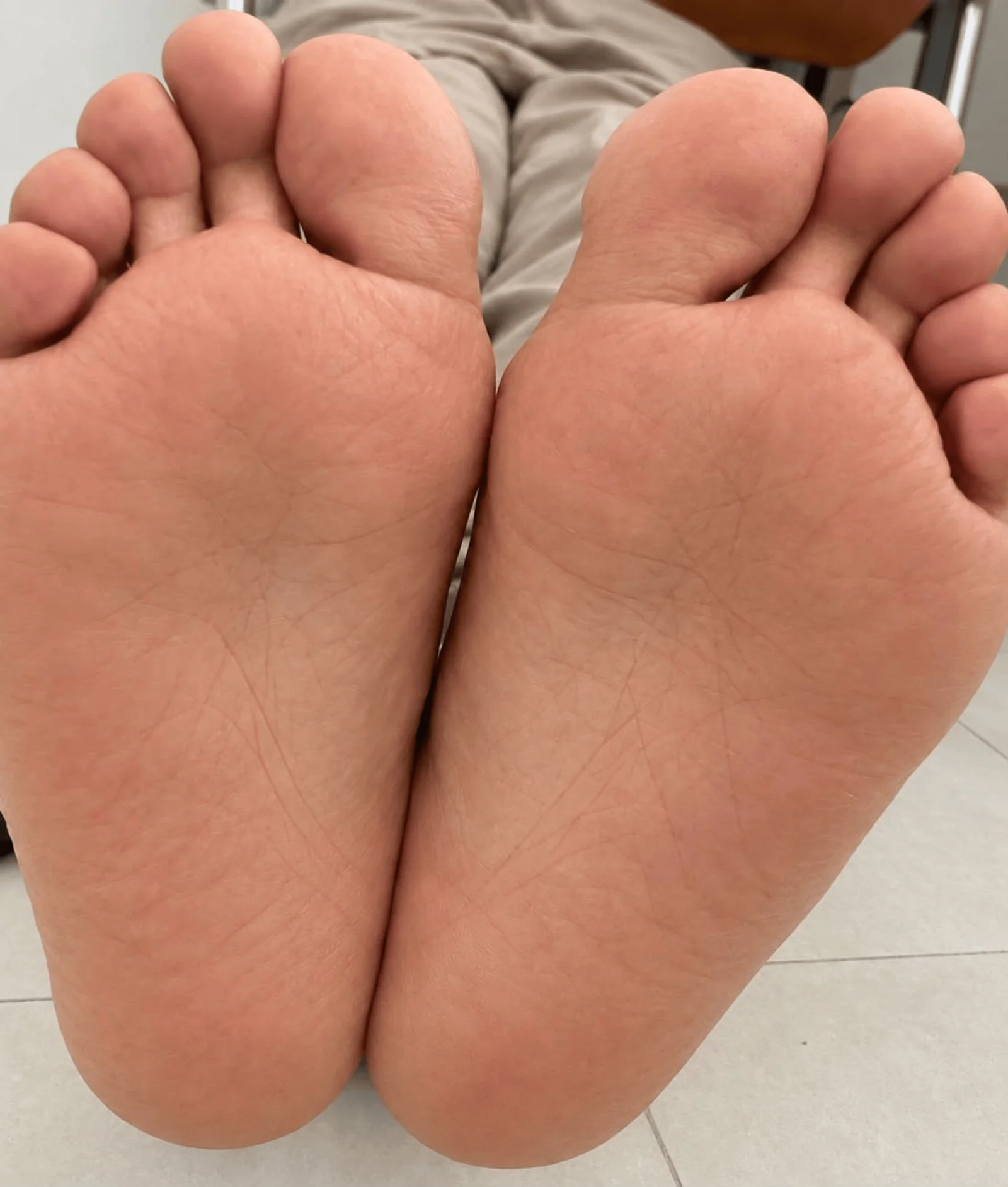
Clinical appearance of the soles after three months of ursodeoxycholic acid therapy, showing complete clearance of plantar keratoderma and erythema.

## Discussion

Palmoplantar keratoderma (PPK) refers to a heterogeneous group of disorders characterized by abnormal hyperkeratotic thickening of the palms and soles. PPKs may be hereditary or acquired and, according to their clinical distribution, may present as diffuse, focal, or punctate forms [1,2]. Diffuse PPK involves relatively uniform palmoplantar hyperkeratosis, focal PPK consists of localized hyperkeratotic areas, typically over pressure points, and punctate PPK is characterized by multiple discrete keratotic papules scattered over the palms and soles [1,2]. Histologically, acquired keratodermas are generally nonspecific, with marked hyperkeratosis of the stratum corneum as the most consistent finding, variably accompanied by acanthosis, parakeratosis, thickening of the spinous and granular layers, and a chronic perivascular inflammatory infiltrate [1].

Acquired PPK has a wide array of underlying etiologies, including drug exposure, chemical agents, nutritional deficiencies, inflammatory and infectious dermatoses, systemic diseases, malignancies, and climacteric or idiopathic forms [1]. Drug-induced PPK is a recognized entity, particularly with targeted anticancer therapies, mainly B-Raf proto-oncogene, serine/threonine kinase (BRAF) inhibitors such as vemurafenib and dabrafenib [7]. Keratoderma climactericum characteristically develops around menopause and predominantly affects pressure-bearing areas of the soles [8]. Aquagenic keratoderma is clinically distinct, with transient whitish papules and accentuated wrinkling following water exposure [9]. Among systemic causes, hypothyroidism and myxedema are recognized associations with acquired PPK [10,11].

Management of acquired PPK relies on identification and treatment of the underlying cause. Symptomatic options include topical keratolytics, retinoids, corticosteroids, debridement, or psoralen plus ultraviolet A (PUVA) therapy; systemic retinoids may be considered in recalcitrant cases [1]. In our patient, the lack of improvement with previous topical therapies, together with the absence of an evident cause, prompted further etiologic investigation, which revealed a cholestatic liver profile with elevated alkaline phosphatase and positive anti-mitochondrial antibodies (AMA). PBC is a chronic autoimmune liver disease characterized by progressive destruction of the small intrahepatic bile ducts, resulting in cholestasis and potentially progressing to fibrosis and cirrhosis [5]. The diagnosis of PBC can be established when at least two of three criteria are present: biochemical evidence of cholestasis, primarily based on alkaline phosphatase elevation; AMA positivity; and compatible histologic findings [12].

Dermatologic manifestations are recognized in PBC and may occasionally precede recognition of the underlying liver disease. In a prospective case-control study by Koulentaki et al. involving 49 patients with PBC, pruritus and xerosis were each reported in 69.3% of patients, dermographism in 57.1%, and melanosis in 46.9%; notably, a dermatologic manifestation was the presenting feature leading to the diagnosis of PBC in 38.7% of patients [6]. Terziroli Beretta-Piccoli et al. described xanthomatous lesions, including xanthelasma and xanthoma striatum palmare, and identified a broader spectrum of reported inflammatory and autoimmune dermatoses, including psoriasis, vitiligo, lichen planus, autoimmune blistering diseases, vasculitis, and neutrophilic or granulomatous dermatoses [13]. However, they emphasized that the available evidence remains limited and does not establish that most of these conditions are more prevalent in patients with PBC than in the general population [13]. Acquired PPK has been exceptionally reported in association with PBC, with only two cases previously described [3,4]. The main clinical characteristics, treatments, and outcomes of these previously reported cases and the present case are summarized in Table 2.

**Table 2:** Comparison of previously reported cases of acquired palmoplantar keratoderma associated with primary biliary cholangitis and the present case. PPK, palmoplantar keratoderma; UDCA, ursodeoxycholic acid; PUVA, psoralen plus ultraviolet A; UVB, ultraviolet B.

Characteristic	Nadhan et al. [3] (2018)	March-Rodriguez et al. [4] (2022)	Present case
Age/Sex	57-year-old woman	57-year-old woman	36-year-old woman
PPK morphology and distribution	Diffuse thickening of the palms and soles with slight erythema	Diffuse, non-transgradient PPK of the palms and soles, more pronounced centrally	Yellowish PPK with moderate erythema; diffuse on the palms and punctate on the soles, with focal coalescence over pressure-bearing areas
Dermoscopy	Not reported	Not reported	Polarized dermoscopy: pink background with round-to-oval yellowish areas, patchy and occasionally confluent scales, no visible vascular pattern, and preserved dermatoglyphic markings
Histopathology/biopsy	Hyperkeratosis, hypergranulosis, and acanthosis; repeat biopsy confirmed PPK	Orthokeratotic hyperkeratosis with mild hypergranulosis and acanthosis	Not performed
Previous skin-directed treatments	Acitretin, topical tazarotene, PUVA, topical bexarotene, and narrowband UVB	Salicylic acid, topical corticosteroids, topical retinoids, and oral acitretin	Topical keratolytics, topical antifungal agents, topical corticosteroids, and topical retinoids; no clinical improvement
Concomitant skin-directed treatment after UDCA initiation	Initially continued; subsequently discontinued after clearance	Not reported	None
UDCA dose	Not reported	1,300 mg/day	900 mg/day (~14 mg/kg/day)
Time to clinical response	Complete clearance within 2 months	Complete resolution after 6 months	Complete clearance after 3 months
Degree of clearance	Complete	Complete	Complete
Duration of follow-up	2 years	2 years	18 months
Long-term cutaneous outcome	Persistent clearance	No recurrence	Sustained clearance with no relapse

Both previous reports involved 57-year-old women with diffuse palmoplantar involvement who achieved complete and sustained clearance after initiation of UDCA for PBC [3,4]. Our observation shares with these reports the acquired nature of the keratoderma, its association with PBC, and the complete and sustained clinical response following UDCA therapy. However, our patient was considerably younger and showed a distinct clinical distribution, with predominantly diffuse palmar involvement and punctate plantar hyperkeratotic lesions, some coalescing into plaques over pressure-bearing areas. In addition, our case provides dermoscopic characterization of the lesions, showing a pinkish background with round-to-oval yellowish areas, patchy and occasionally confluent scales, and absence of a visible vascular pattern. However, given the absence of dermoscopic descriptions in the previously reported cases, the specificity of these findings for PBC-associated PPK remains unknown. The absence of histopathological confirmation remains a limitation. However, the clinical presentation and diagnostic work-up support the diagnosis of acquired PPK.

UDCA is the established first-line treatment for PBC and improves cholestatic liver biochemistry and long-term clinical outcomes [5]. Improvement or complete resolution of the associated PPK following UDCA therapy has been observed in both previously published cases and in our patient [3,4]. In our case, complete clearance of the palmoplantar lesions after three months of treatment occurred together with normalization of liver biochemical tests, with no relapse during 18 months of follow-up. Although this temporal relationship does not establish causality, it supports the possibility of a pathogenic link between PBC and acquired PPK.

The pathophysiological link between PBC and acquired PPK remains unclear. Nadhan et al. proposed a reactive mechanism, based on the observation that acquired PPK associated with systemic diseases may regress after treatment of the underlying disorder [3]. The similar clinical course observed in the two previously reported cases and in our patient supports this hypothesis, although the temporal association does not establish causality [3,4]. Given that PBC is characterized by both immune dysregulation and cholestasis [5], either process could theoretically contribute to the development of cutaneous changes. Altered bile acid homeostasis may influence cutaneous immune and inflammatory pathways, providing a potential link between cholestasis and skin manifestations; however, its role in palmoplantar hyperkeratosis remains unknown [14]. Additional indirect observations support a possible immune-mediated cutaneous expression of PBC.

Iwadate et al. reported palmoplantar pustulosis in a patient with PBC and Behçet’s disease and proposed T-cell-mediated interactions involving keratinocytes and biliary epithelial cells [15]. Although palmoplantar pustulosis is distinct from PPK and Behçet’s disease is a potential confounding factor, this report provides indirect support for a localized immune-mediated palmoplantar manifestation of PBC. Similarly, erythema nodosum has been reported in early PBC, suggesting a possible immune-mediated association [16]. Whether the cutaneous response to UDCA reflects improvement of the underlying cholestatic and immune disturbance rather than a direct effect on the skin remains uncertain. Taken together, these observations suggest that acquired PPK may represent a rare reactive cutaneous manifestation of PBC; however, the very small number of reported cases and the lack of a demonstrated mechanism preclude any definitive causal conclusion.

## Conclusions

Acquired PPK associated with PBC is an exceptionally rare clinical presentation. This case highlights the importance of considering an underlying systemic disorder in patients with otherwise unexplained or treatment-refractory acquired PPK, including hepatobiliary disease when supported by clinical or laboratory findings. The complete clearance of the palmoplantar lesions alongside normalization of liver biochemical abnormalities after UDCA therapy, with no relapse during 18 months of follow-up, supports a possible association between the two conditions, although causality remains unproven. Recognition of such uncommon associations may facilitate earlier diagnosis and appropriate multidisciplinary management.
